# Open Science, Open Data, and Open Scholarship: European Policies to Make Science Fit for the Twenty-First Century

**DOI:** 10.3389/fdata.2019.00043

**Published:** 2019-12-10

**Authors:** Jean-Claude Burgelman, Corina Pascu, Katarzyna Szkuta, Rene Von Schomberg, Athanasios Karalopoulos, Konstantinos Repanas, Michel Schouppe

**Affiliations:** Open Science, DG Research and Innovation, European Commission, Brussels, Belgium

**Keywords:** open science, open data, open access, open scholarship, European science policies, artificial intelligence

## Abstract

Open science will make science more efficient, reliable, and responsive to societal challenges. The European Commission has sought to advance open science policy from its inception in a holistic and integrated way, covering all aspects of the research cycle from scientific discovery and review to sharing knowledge, publishing, and outreach. We present the steps taken with a forward-looking perspective on the challenges laying ahead, in particular the necessary change of the rewards and incentives system for researchers (for which various actors are co-responsible and which goes beyond the mandate of the European Commission). Finally, we discuss the role of artificial intelligence (AI) within an open science perspective.

## Open Science Is Science for the Twenty-First Century

Open science as such is not a new concept, and many terms have been used to refer to the transformation of scientific practices, such as Science 2.0 (Burgelman et al., 2015; Szkuta and Osimo, 2016). Multiple approaches that exist to the transformation to open science (Fecher et al., 2015) are all rooted in the tradition of openness of science. The European Commission started using the term “open science” as a result of the public consultation on Science 2.0 Science in Transition in 2014 (European Commission, 2015). An overwhelming 42% of the nearly 500 respondents to this consultation (among which large scientific organizations or associations) preferred the term “open science” over alternatives such as Science 2.0. The European Commission respected this choice of the term, although the European policies are directed toward “open scholarship,” as “open scholarship” reflects the inclusion of the humanities in the equation as well as emphasizing the open input side to science in the form of open collaboration and active data and knowledge sharing *prior* to publishing and other scientific open outputs. Horizon Europe, the new EU Framework Programme for Research and Innovation, will promote open science in the full meaning of open scholarship. In this vein, Von Schomberg (2019) defined open scholarship as sharing knowledge and data as early as possible in the research process in open collaboration with all relevant knowledge actors. In this article, we employ the term “open science” in this broad meaning.

Open science in essence refers to the transformation that science is undergoing due to globalization and ICT—just like any other sector in society—and it is therefore very likely that in the long term, the adjective *open* should not be necessary as science will be open by default. An early and well-known example of open science, from the pre-Internet stage is the Human Genome Project that started in 1990. The data on the human genome were widely shared among the scientific community in the course of the project while a moratorium on publishing was kept to encourage optimal collaboration. Because of this openness, they were able to decode the human genome in <15 years. Open science (or in fact, open scholarship) has shifted the prime focus of researchers away from publishing toward knowledge sharing.

ICT is critically enabling open science, but open science is more than a technology-driven change. Several elements of the life cycle of research need to be in place. One of the most important ones for open science to succeed is open data. The latter is a condition *sine qua non* for reproducibility and scientific progress. Open data speed up the research process by facilitating re-use and enriching datasets (King, 2011; Piwowar et al., 2011; Whitlock, 2011) while making the most of (public) investment in the production of research data. Opening up data enables to detect false claims and inaccuracies and allows for replicability tests (e.g., Ioannidis and Khoury, 2011). In essence, it allows more use of the same investment and thus more scope for discovery, in particular, for addressing crosscutting research questions like most of the big challenges that affect the world (UN Sustainable Development Goals^1^). Finally, it gives credit to data creators increasing their citation rate and therefore their research impact (Piwowar et al., 2007). Opening up research data also impacts the social web (Tenopir et al., 2011; Wallis et al., 2013; Peters et al., 2016).

The cases of the Ebola and Zika epidemics show on the one hand the advantages of open science and on the other side researchers' dilemmas. The many deaths due to the Ebola epidemic in West Africa during 2014–2016 could have been prevented (Knobloch et al., 1982) using existing public knowledge. On the verge of Ebola epidemics, researchers took the initiative to share data concerning the virus early on with the result that an experimental vaccine became quickly available^2^. The World Health Organization [World Health Organization (WHO), 2015] seeks a paradigm shift in the approach to information sharing in public health emergencies from one limited by embargoes set for publication timelines to open sharing using modern fit-for-purpose prepublication platforms. Researchers, journals, and funders will need to engage fully for this paradigm shift to occur. The WHO acknowledged that patents on natural genome sequences could be inhibitory for further research and product development and wants research entities to exercise discretion in patenting and licensing genome-related inventions so as not to inhibit product development and to ensure appropriate benefit sharing. The organization also wants scientific publishers not to penalize, but to encourage or mandate public sharing of relevant data. Zika was the next major emerging public health issue, following the Ebola example, which was encountered with effective initiatives based on open scholarship. The National Institutes of Health in the United States now requires grantees to make large-scale genomic data public by the time of publication at the latest.

Shared use of data goes beyond one discipline, expanding the scope of research and diversifying perspectives (Fischer and Zigmond, 2010). It also allows for creation of new (meta) knowledge (Evans and Foster, 2011). Still, sharing data is impeded by lack of formal recognition as data citations are not yet standard practice (Costas et al., 2013) and by resistance from researchers who think that open data will jeopardize their individual publishing trajectory and impact (journal impact factors and citations; Scheliga and Friesike, 2014).

Changing the reward and incentive system for researchers is a key open science challenge and a broader issue for which primarily the responsibility lies in the scientific community (universities and funders). This includes making open science practices rewardable and fundable as well as the employment of specific indicators for researchers' engagement with open science. A change of the reward and incentive system can only be stakeholders-driven, and it has to be bottom-up. This change also includes changing mind-sets of researchers to open up and share data and “seduction” to make open science easy, useful, and affordable^3^.

## European Holistic Policy to Open Science

To make sure that Europe's scientific eco system will be fit for the new modus operandi of open science, the European Commission in a co-design and co-development mode with the key scientific stakeholders developed a holistic policy to promote the changes needed for making open science a European reality^4^. The European Commission's approach has been embraced by several funders and institutions and used as a model for their own policies.

It also inspired other continents to issue similar policies of statements, for instance, the calls for the research community to work together to realize “open science by design” (National Academies of Sciences, Engineering, and Medicine, 2018), initiatives (such as the Australian research data infrastructure initiative^5^ and is now being translated to the supranational level; G7 work in Open Science Working Group^6^), and OECD work on Enhanced Access to Data and Models (OECD, 2006) and Business models for sustainable research data repositories (OECD, 2017), the African Research Cloud (ARC), and UNESCO.

The kickoff moment was the publication of the then new commissioner for research and innovation C. Moedas' vision for Europe “Open Innovation, Open Science, Open to the World.” For the first time, a commissioner made addressing the changes in the science system one of its key priorities (European Commission, 2016a).

Right from the start, the then director-general (DG) of the Commission for Research and Innovation, RJ Smits, wanted the Commission to lead by example by making^7^ open access to peer-reviewed publications mandatory and encouraging open access to research data for those projects funded by the EC. Access to and re-use of research data generated by Horizon 2020 and subsequent projects will be improved, and access will be maximized. In Horizon Europe, research data will be open by default while taking into account the need to balance openness and protection of scientific information, commercialization and Intellectual Property Rights, privacy concerns and security, following the principle “as open as possible, as closed as necessary.” Data management plans (DMP) will become mandatory, even if not making research data open. The requirement for responsible data management will be separated from the requirement for providing open access to research data. Emphasis will be placed on supporting as much as possible the proliferation of data that are findable, accessible, interoperable, and re-usable (FAIR). Finally, the use of trusted or certified repositories and infrastructures like the European Open Science Cloud (EOSC) will be required for research data in some Horizon Europe work programs.

Open access to publications is already mandatory in Horizon 2020. Researchers need to deposit a copy of the published version or final peer-reviewed manuscript in a repository of their choice at the latest on publication and ensure open access to the publication *via* the repository within 6 months of publication or 12 months in case of the social sciences and humanities. Repositories will continue to play a key role in the Commission's policy on open access in Horizon Europe.

In 2018, the Commission decided to support Plan S' ambitions to move forward toward open access. Plan S was launched in September 2018 under the auspices of the president of Science Europe, Marc Schiltz, and former DG Robert-Jan Smits, with the aim that “*After 1 January 2020, scientific publications resulting from research funded by public grants provided by national and European research councils and funding bodies must be published in compliant Open Access Journals or Platforms.”*

As a supporting organization, the Commission is committed to accelerating the full transition toward open access to scientific publications and will continue to work in a concerted effort with cOAlition S members (research funders committed to Plan S) to ensure a consistent approach. Actions are now ongoing to complete the transition to open access in line with Plan S. In Horizon 2020, the Commission is enforcing its mandate on open access and supporting Plan S implementation without making legal changes (e.g., Open Research Europe platform, highlight of existing OA requirements, monitoring, and sanctioning). The implementation of Plan S in legal texts will be in Horizon Europe (Regulation/MGA), including Plan S principles, such as Intellectual Property Rights retention, open licenses, immediate open access, or the further requirements for repositories and OA venues.

The Commission has also moved beyond open access to promote and advance open science. Open science practices will be embedded in selected Horizon Europe work programs, depending on the scientific discipline and their particular focus. Incentives will include eligibility of costs for practices such as early sharing of work or sharing research output beyond publications and data. The Commission is already exploring ways to ensure that researchers engaging in open science practices will be rewarded for that, and new-generation metrics such as data citation may be introduced to provide a more nuanced understanding of the wider impact of research publicly funded by the EC.

Despite the many advantages of having open research data, there seems to be less awareness of what the data science revolution will imply in terms of costs for implementing measures that would facilitate the change. Governments support building new research infrastructures, but the resources for maintenance including the growth of data exponentially growing needs for data hosting and stewardship are not aplenty (European Commission, 2016b). Both institutional and thematic repositories host data and develop their own strategies. Yet, the uncoordinated efforts result often in discrepancies between repositories and the lack of synergies. There are two (non-budgetary) approaches proposed to solve this. One is technical, that is, FAIR guidelines, while the second is using FAIR as an important enabler in a federated infrastructure.

FAIR^8^ data (data that are findable, accessible, interoperable, and reusable) (Wilkinson et al., 2016) play an essential role in the objectives of open science to improve and accelerate scientific research to increase the engagement of society and to contribute significantly to economic growth. Without FAIR research data, open science is simply impossible.

The European Open Science agenda contain the ambition to make FAIR data sharing the default for scientific research by 2020. To support the implementation of the FAIR data principles^9^ in Europe and beyond with tangible and actionable recommendations, the Commission established a FAIR Data expert group^10^. The recommendations^11^ from that expert group describe a broad range of changes (policy, cultural, and technical) to turn FAIR into reality in Europe (European Commission, 2018): FAIR Digital Objects to enable discovery, citation, and reuse; data services to support FAIR; interoperability frameworks to incorporate research community practices; a distributed, federated infrastructure to unlock the potential of analysis and data integration; skills for data science and data stewardship; incentives for open science (metrics and indicators); and funding for FAIR to bring strong return on investment.

The annual opportunity cost of not having FAIR research data (European Commission, 2019a,b) is estimated to be at least €10.2 bn for the European scientific system. In addition, it is estimated that not having FAIR would also result in another €16 bn annual opportunity cost for the wider research and innovation system.

Aiming to increase the coherence and interoperability of FAIR assessment frameworks, the Commission initiated work under the Research Data Alliance (RDA) “FAIR Data Maturity Model” Working Group^12^ to develop a set of core assessment criteria for FAIRness and a generic and expandable self-assessment model for measuring the maturity level of a dataset. The group has brought together more than 100 representatives of stakeholders from different scientific and research disciplines, the industry and public sector, who are interested in the FAIR principles and in the creation of assessment methodologies for evaluating their real-life uptake and implementation (Sansone et al., 2019).

Europe indeed faces considerable problems of non-interoperable services and research data and limited cross-disciplinary access to these research data. It is difficult for researchers to organize and store their own data so that it is usable for themselves at a later date, let alone usable for other researchers in the long term. Notwithstanding these challenges, the elements needed to create a “commons for scientific research data” are already in place, but they are lost in fragmentation across member states and across different scientific communities (European Commission, 2016a). The process toward an EOSC “commons for scientific data” is community-driven and multi-level, that is (multi-)national, regional (Europe), and global (Budroni et al., 2019). In 2018, the European Commission has initiated the process that leads to the creation of an “Internet for science,” on principles of minimal governance, maximum freedom to implement, globally interoperable and accessible, and globally embedded in a “commons” based on scientific data (European Commission, 2016a). The term “cloud” in European Open Science Cloud^13^ is understood as a metaphor for a service that aims to be seamless and in support of the idea of a commons: making it possible, on equal conditions, for 1.7 million researchers in Europe to store, share, and re-use data across nations and scientific disciplines through the open science cloud and without leaving their desk. EOSC is not a cloud “made in Brussels” and will not be built on a “green field” (considerable past infrastructure investments 10 billion euros per year by the EU and Member States over the last two decades and existing know-how). GO FAIR is a bottom-up international approach for the practical implementation of the European Open Science Cloud as part of a global Internet of FAIR Data & Services^14^.

EOSC is facing complex governance issues, and a strong but flexible “federal” governance model would be needed based on trust and increasing mutuality, representativity, proportionality, accountability, inclusiveness across disciplines and countries, and transparency. The new EOSC governance framework^15^ fulfills these principles and illustrates perfectly some of the functions of an early-stage start-up board (such as key strategic decision making and oversight, accountability) as well as the entrepreneurial attitude needed for the creation of EOSC (“build it and scale it up”).

## AI for Better Science

Even if we can say that toward the end of the second decade of the twenty-first century the idea of data-driven science has been accepted as the new reality research, the EC does believe that this is only the start of a deeper change. The science system is in “landslide transition from data-sparse to data-saturated” (European Commission, 2016b). The quantity of data produced is already growing exponentially. Ninety percent of the world's data today has been generated just over the last 2 years (2.5 quintillion bytes of data per day)^16^.

But with the Internet of everything (humans and artifacts), all that happens on the globe and beyond will become somehow a data point and therefore fit for research. Today, it is quasi-standard practice to use TDM technologies for data analytics and processing on the cloud. Online collaborative tools (Pascu et al., 2007) are the new lab science as a “flow” of beta products become accepted in some disciplines.

AI will push again the frontier of getting knowledge and making meaning out of it (Elsevier, 2018). This is comparable to the impact of the introduction of PC and the Internet 40 years ago. The potential is virtually “limitless.” In medicine, for instance, AI can help identify new genes related to cancer^17^, spot indicators of eye disease^18^, and recommend how patients should be referred for care or to find peers working on the same treatments. In the long run, it could be extended to other areas of knowledge^19^.

AI has already shown potential to accelerate the data discovery and data analysis and to extract knowledge out of research artifacts^20^ (Sinha et al., 2015; Wang, 2019). AI technologies can act as a catalyst for further scholarly discussion^21^ and change the way research contributions are recognized (Piwowar, 2013), for example, in the peer-review process^22^^,^^23^.

The single most important challenge remains whether AI could have a key role in determining the originality of research, one of the cornerstones on which science is built. Reproducible research makes for more efficient and reliable science. Evidence points to the fact that over 70% of researchers fail to reproduce other peers' research^24^. One reason is that the experimental setup is rarely described (only about half of papers include code that was built)^25^. Expectations are for AI to improve the credibility of the research^26^ and efficiency of research by “opening up the models” (Dodge et al., 2019).

## What Is Still Missing?

The Ebola case of public health emergencies provides an inspiring model for how global research collaborations can help address the societal challenges of our times. Such cases should not be an exception but the norm. However, to make open science the norm, as the dramatic cases of Ebola and Zika illustrate, open science policies that relate to the core of the work of researchers need to be implemented, addressing the necessary change of the rewards and incentive system for researchers. This implies as the case of emerging public health emergencies illustrate that the importance of publishing in major scientific journal will be relativized in the context of a full operational open science, and other research outputs as open data, open software, and so forth will become important. Open research outputs will be available prior to publication rather than postpublication.

AI technologies have the potential to foster an inclusive science community. But a good AI is dependent on the variety and quality of data. Open data can play a key part for AI algorithms and machines to function and produce good outcomes^27^.

Transition to open science is a multidimensional and multistage process. There is value and risk of being a first mover, but there is higher risk of being a follower. The European Commission has taken various steps in initiating this transition, but all stakeholders must get on board to take mutually reinforcing steps to advance open science policy and its implementation (Euroscientist, 2015). After all, the EC is, rightfully so, not even competent on many domains where open science policies should be developed (e.g., rewards and incentives).

## Author Contributions

All authors listed have made a substantial, direct and intellectual contribution to the work, and approved it for publication.

### Conflict of Interest

The authors declare that the research was conducted in the absence of any commercial or financial relationships that could be construed as a potential conflict of interest.

## Footnotes

^1^https://sustainabledevelopment.un.org/?menu=1300

^2^WHO then requested in 2015 that the sharing of research data and results become the global norm during public health emergencies and mediated agreements with publishers to share information prior to publication.

^3^https://www.open-science-conference.eu/wp-content/uploads/2019/03/Eva-Mendez.pdf

^4^For instance the Open Science Policy Platform established by the European Commission, https://ec.europa.eu/research/openscience/index.cfm?pg=open-science-policy-platform

^5^https://www.rds.edu.au/single-post/2018/03/15/Developing-a-%E2%80%98National-Research-Data-Cloud%E2%80%99-for-Australian-Researchers-Release-from-the-Department-of-Education

^6^The G7 Open Science Working Group (OSWG) has been established in 2016 by the G7, https://www8.cao.go.jp/cstp/kokusaiteki/g7_2016/ 20160517communique.pdf. Its overarching ambition is to share expertise, exchange best practices and develop synergies on Open Science paradigms. The OSWG is jointly chaired by the European Commission and Japan.

^7^An initial open access pilot run in FP7 followed by a flexible open access for research data pilot in Horizon 2020 called “ORD pilot” aimed selected areas of Horizon 2020. The ORD pilot was extended gradually from several selected areas to all thematic areas of the Horizon 2020 Research and Innovation Programme.

^8^The FAIR data principles define a minimal set of community-agreed “aspirational” guidelines for the publication of digital resources such as datasets, code, workflows, and research objects, to achieve a state of “FAIRness” (Wilkinson et al., 2018).

^9^https://www.go-fair.org/fair-principles/

^10^http://ec.europa.eu/transparency/regexpert/index.cfm?do=groupDetail.groupDetail&groupID=3464

^11^European Commission's Expert Group on FAIR Data published the interim report ‘Turning FAIR Data into Reality’ and the interim ‘FAIR Data Action Plan’ on 11 June 2018 at the Second EOSC Summit in Brussels.

^12^https://www.rd-alliance.org/groups/fair-data-maturity-model-wg

^13^In November 2018, under the Austrian Presidency, the European Commission launched the European Open Science Cloud (EOSC) at the University of Vienna, Vienna Declaration on EOSC.

^14^https://www.go-fair.org/

^15^The EOSC Governing Board (GB) gathers representatives from the EU member states, the associated countries and the European Commission; the EOSC executive board (EB) is composed of representatives of pan-European research organizations and few independent experts.

^16^http://www.sciencedaily.com/releases/2013/05/130522085217.htm; https://www.forbes.com/sites/bernardmarr/2018/05/21/how-much-data-do-we-create-every-day-the-mind-blowing-stats-everyone-should-read/#686d324660ba

^17^For instance https://www.nature.com/articles/s41467-019-08797-8 and Sampo Pyysalo et al. ‘LION LBD: a Literature-Based Discovery System for Cancer Biology, https://academic.oup.com/bioinformatics/advance-article/doi/10.1093/bioinformatics/bty845/5124276

^18^Google Deepmind algorithm show a correct referral rate of 94 per cent for over 50 eye disorders, https://sciencebusiness.net/news/deepminds-ai-doctor-predicted-transform-eye-disease-diagnosis

^19^https://techcrunch.com/2017/01/23/chan-zuckerberg-initiative-meta/?guccounter=1

^20^Semantic Scholar analyses medical publications (started with computer science in 2015, the service has been scaled to biomedicine) and extracts important features using machine learning techniques (context understanding), https://www.semanticscholar.org; https://www.economist.com/science-and-technology/2017/10/19/a-better-way-to-search-through-scientific-papers?zid=291&ah=906e69ad01d2ee51960100b7fa502595

^21^Tools like IrisAI, https://iris.ai/, Elsevier's Euretos AI platform or IBM's Automated hypothesis generation system, http://scholar.harvard.edu/files/alacoste/files/p1877-spangler.pdf

^22^Several major publishers have already started to integrate AI in their digital platforms to address quality control and reviewer identification, or for checking for potential conflicts of interests between editors, reviewers and authors e.g., Frontiers, https://blog.frontiersin.org/2018/12/14/artificial-intelligence-peer-review-assistant-aira/

^23^https://projectaiur.com/#value-growth

^24^Nature survey https://www.nature.com/news/1-500-scientists-lift-the-lid-on-reproducibility-1.19970

^25^https://www.wired.com/story/artificial-intelligence-confronts-reproducibility-crisis/

^26^Center for Open Science (COS) large-scale collaboration program with DARPA (SCORE program) to create a database of research claims and results from published papers in the social and behavioral sciences. The database will be “enhanced” with evidence from other sources, such as citations or indications on whether the data were openly accessible, and used to train AI algorithms to score the claims for their likelihood of being reproducible findings, https://cos.io/about/news/can-machines-determine-credibility-research-claims-center-open-science-joins-new-darpa-program-find-out/.

^27^An example of an AI project using Open Data for crime prevention (https://www.policyconnect.org.uk/appgda/research/crime-prevention-through-artificial-intelligence) resulted in reducing burglaries by 33% and violent crimes by 21%.
